# Outcomes and recovery timelines of 249 patients with Parsonage–Turner syndrome from a major tertiary care center

**DOI:** 10.1016/j.jseint.2026.101764

**Published:** 2026-07-22

**Authors:** Jonas Vorbau, George E. Sayegh, Zachary Sigman, Andrew K. Chow, Hunter Gutting, Abigail E. Bass, Evie O'Brien, Frederick J. Pimental, Chelsea Swei, Alexa Cohen, Sophia I. Blaine, Marco T. Di Stefano, Michael G. Young, Zachary A. Panton, Zachary S. Silber, Thomas A. Gagliardi, Mark P. Cote, Ryan Lohre, Bassem Elhassan, Alireza Gholipour, Augustus D. Mazzocca

**Affiliations:** Enable Education and Research Facility, Division of Sports Medicine, Department of Orthopaedic Surgery, Massachusetts General Brigham Hospital, Harvard Medical School, Boston, MA, USA

**Keywords:** Orthopedics, Parsonage–Turner syndrome, Shoulder, Nerves, Recovery, Outcomes, Neuralgic amyotrophy, Brachial neuritis

## Abstract

**Background:**

Parsonage–Turner syndrome (PTS) is a neuromuscular disorder characterized by sudden, severe shoulder and arm pain followed by weakness. Recovery time varies considerably, creating prognostic uncertainty. This study used a tertiary care health system database to characterize recovery trajectories and identify demographic factors associated with recovery duration.

**Methods:**

Patients from Mass General Brigham system were identified using an Enterprise Data Warehouse query for diagnoses of “Parsonage Turner Syndrome,” “neuralgic amyotrophy,” and “brachial neuritis” from a dataset of 4,800,000 patients from January 2016 to March 2025. Of 2,552 identified patients, 249 met strict clinical criteria for confirmed PTS. Key time points were extracted from clinical notes and Kaplan–Meier survival analyses were performed to estimate time to recovery.

**Results:**

The cohort included 249 patients (60.2% male) with a median age at symptom onset of 54.2 years (range: 19.1-88.6 years). Kaplan–Meier analysis demonstrated a cumulative median time to recovery of 72 months, whereas median recovery timelines of those with documented recoveries were shorter (functional recovery = 307.5 days; 100% recovery = 400 days). Neither age nor sex impacted recovery timelines (log-rank *P* = .68 and *P* = 1.00, respectively).

**Conclusion:**

Recovery from PTS was variable. Only 56 patients contributed recovery events to Kaplan–Meier analysis, reflecting the difficulties in ascertaining follow-up recovery data in a retrospective chart review. Neither age nor sex was associated with recovery time. These findings illustrate the demographics and range of recovery timelines observed in a large cohort while underscoring the need for prospective studies to define recovery with confidence.

Parsonage–Turner syndrome (PTS), also clinically referred to as neuralgic amyotrophy^17^^,^^47^ and brachial neuritis (BN),^35^^,^^45^^,^^47^ is a rare inflammatory neuropathy characterized by sudden, severe shoulder and arm pain followed by patchy paresis and muscle atrophy from disrupted brachial plexus innervation.^11^^,^^15^^,^^16^^,^^28^^,^^30^^,^^36^^,^^44^^,^^50^ The initial pain typically subsides over days to weeks and is succeeded by profound muscle weakness, which can endure for months to years.^3^^,^^18^^,^^26^^,^^41^^,^^42^

Incidence rates vary, with reported rates including 1.64 cases per 100,000,^5^ 10.3 per 100,000,^20^ and 1 per 1,000.^49^ Despite increasing awareness, PTS continues to be underdiagnosed and undertreated, as it is often misdiagnosed as cervical radiculopathy.

This variance in patient demographic and disease etiology contributes to the lack of standardization in care and documentation of PTS. Precise etiologic causes of PTS are difficult to identify and poorly defined. Antecedent events are often associated with PTS onset, including viral infections, immunizations,^3^ genetic factors, traumatic injuries, and surgical interventions. Thirty percent to 70% of PTS cases are reported to have an antecedent event,^43^ though Bauer and Resnick caution that antecedent events may not indicate a causal relationship.^4^ Past literature has found PTS to have a higher incidence in males, with incidence rates ranging from 11:9 to 11.5:1.^12^ The dominant hand is affected in 58% of cases, of which 60% were right-handed.^32^

The recovery timeline and outcomes of those suffering from PTS are not well defined.^11^^,^^29^ Previous research by Tsairis followed 99 patients, reporting symptomatic improvements beginning in 1 to two months, and complete functional recovery lasting up to three years or longer. The same study reported that approximately 89% of patients regained full functionality by 3 years, though electromyography (EMG) evidence of complete nerve reinnervation was not systematically assessed.^48^ Variance in recovery time results in uncertainty in disease prognosis for physicians and patients alike.

A 2024 systematic review of 25 studies comprising 950 PTS patients highlighted substantial gaps in the literature, including inconsistent diagnostic criteria, variability in reported outcomes, and limited long-term follow-up data.^2^ Given the inherent variability of patient notes, imaging interpretation, and patient histories and comorbidities, it is impossible to standardize patient selection for larger dataset analysis. These limitations underscored the need for a large-scale analysis with clearly defined diagnostic criteria. To address this, this retrospective study used Massachusetts General Brigham's patient records to evaluate the diagnosis, treatment, and recovery course of PTS patients. With clearly defined inclusion criteria, this study aimed to maximize diagnostic and recovery standardization across the cohort. We hypothesized that age and sex would have a meaningful impact on recovery duration as these demographic factors may reflect biological differences in nerve damage recovery. Understanding the impact of these factors on the outcomes of patients with PTS is of clinical importance as it may allow clinicians to better provide individual management of patients.

## Materials and methods

### Patient extraction

This study was approved under IRB approval (#2022P003141). A retrospective chart review of the Massachusetts General Brigham Enterprise Data Warehouse, which contains 11,100,000 data points and 4,800,000 patients, was performed to identify patients with PTS. A query pulled patients with diagnoses “Parsonage Turner Syndrome,” “neuralgic amyotrophy,” and “brachial neuritis” with the following International Classification of Diseases (ICD)-10 codes: G54.5, M54.10, M54.12, from January 1, 2016, through March 31, 2025.

### Patient screening/data extraction

Two reviewers independently screened each patient's medical records to confirm PTS. Conflicts were resolved by a third reviewer (J.V.), and their resolution was final. Because the conflict resolutions were recorded as a single consensus rather than retained separately for each reviewer, inter-rater agreements and kappa statistics could not be calculated.

The inclusion of a patient was determined against a predefined inclusion checklist applied to every patient. A confirmed case of PTS required all of the following: (1) a clinical presentation of abrupt onset of shoulder pain; (2) this shoulder pain had to have been followed by severe shoulder weakness, and the presence or absence of a winged scapula was extracted; (3) and minimal to no recovery within the first two weeks of presentation. These clinical presentations were supported by imaging. EMG was used as the gold standard to include PTS and exclude conditions with similar presentations. When EMG was not available, magnetic resonance imaging (MRI) imaging consistent with PTS was used as supporting evidence. When patients were seen outside of the Massachusetts General Brigham system but had a confirmed PTS from another system, they were included in this cohort for demographic characteristics but were not included in the outcomes analysis. The diagnosis of patients excluded from the study was recorded. Each patient was only included once, and recovery data were assessed for a single episode. A spreadsheet was used for data extraction.

Given the inherent variability in documentation between physicians, specific criteria for recovery were defined. These included functional recovery, which was defined as patient self-reported ≥80% recovery, as noted by the physician's note, or a return to activities of daily living, and 100% recovery, defined as complete return to strength and range of motion. Key dates, including the date of birth, date of onset, date of diagnosis, date of functional recovery, and date of 100% recovery, were extracted. Additionally, sex, dominant hand, extremity affected, potential causes, interventions/treatment, diagnostic methods/secondary testing, and the specialty of the treating physician were extracted and organized. These specialties were grouped as non-surgical specialties including internal medicine, neurology and physical medicine, and rehabilitation and a surgical group composed of patients seen by orthopedics. Some patients in the neurology group were referred to neurosurgery to confirm a PTS diagnosis, but their care was under the neurologist, and these patients were thus grouped into the non-surgical group. When only a month and year were given, the 15th of the month was used as the date of onset, date of diagnosis, and date of recovery.

### Statistical analysis

Descriptive statistics, including median and range for continuous variables and frequency and proportion for categorical variables, were calculated to characterize the cohort. Time to recovery was analyzed using Kaplan–Meier methods, with recovery defined as ≥ 80% and unrecovered patients censored at their last follow-up. Estimates of median time to recovery were obtained from restricted cohorts (cumulative, n = 216; sex, n = 216; age, n = 178) to account for varying lengths of follow-up time. Of the 249 confirmed cases, 33 lacked a usable onset and diagnosis date, most commonly because the diagnosis was made by an out-of-network provider and the patient was seen in the Massachusetts General Brigham system for an unrelated medical event. These patients were included in the descriptive cohort but excluded from the outcomes analysis. Patients who failed to reach a minimum of functional recovery at the time of their last visit were censored. Of the 216 patients in the restricted cumulative and sex cohorts, 56 had a recovery event, and 160 were censored. The restricted age cohort was smaller (n = 178) because only 186 patients had an age at onset, and an additional 8 patients were excluded because they were seen out of the Massachusetts General Brigham system. Separate estimates were obtained based on patient sex and age. Differences between strata were compared using the log-rank test and 95% confidence intervals (CIs) were obtained for median recovery timelines. Because the estimated median recovery time may be influenced by heavy censoring and small numbers of patients remaining at risk during extended follow-up, cumulative recovery probabilities and corresponding 95% CIs were estimated at 12, 24, 36, 48, and 60 months. Associations between covariates, including age and sex, and recovery were evaluated using Cox proportional hazards regression. Finally, the distribution of follow-up times among censored patients was examined graphically overall and stratified by sex to evaluate potential differences in follow-up. All analyses were carried out in R (R Core Team, http://www.r-project.org) with the *survival*^46^ and *survminer*^23^ packages.

## Results

**Figure 1:**
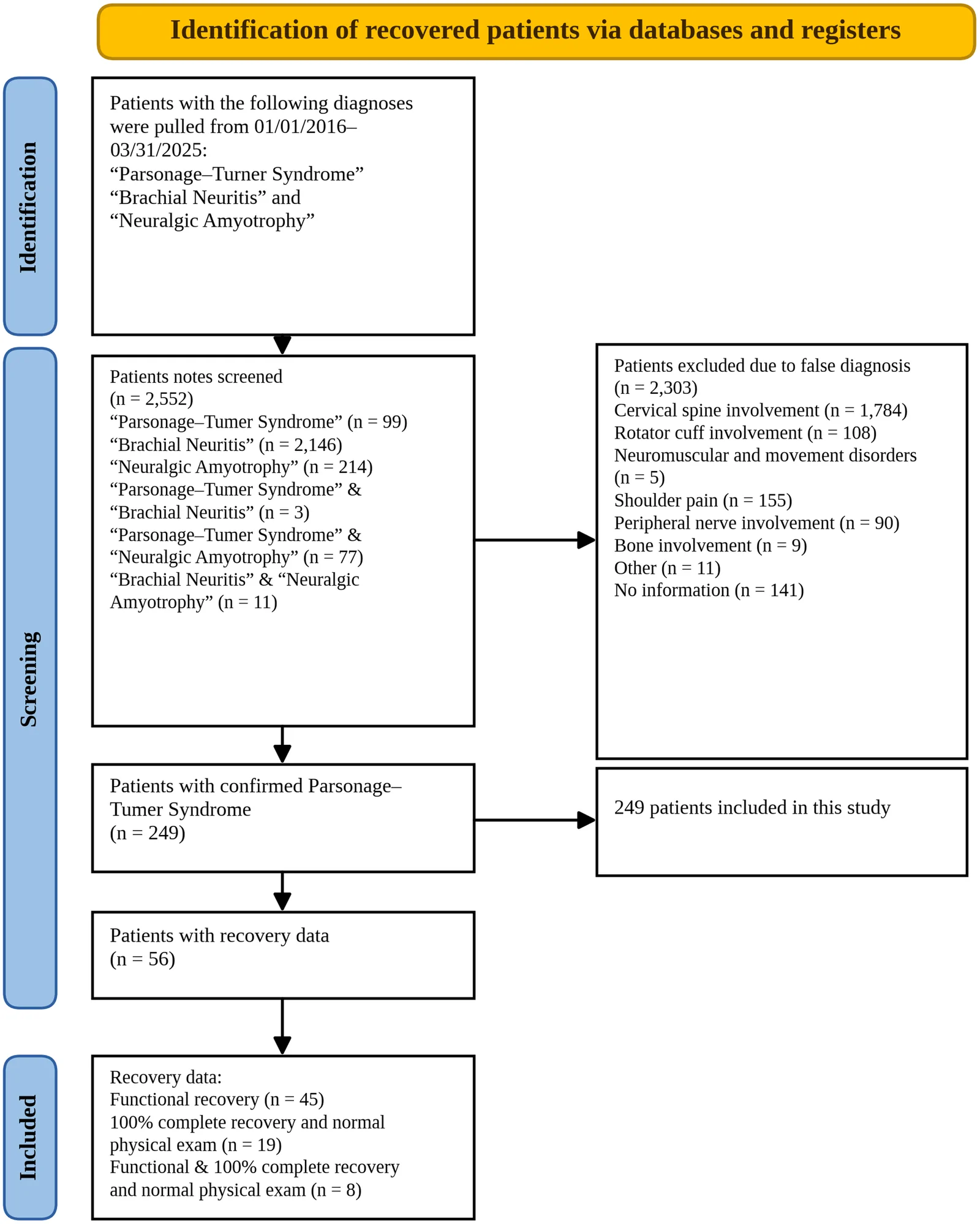
PRISMA flow diagram for the identification and screening of Parsonage–Turner syndrome cases (2016–2025) admitted to MGB. *PRISMA*, Preferred Reporting Items for Systematic Reviews and Meta-Analyses; *MGB*, Massachusetts General Brigham.

From 4.8 million patients in the Enterprise Data Warehouse query, 2,552 unique patients were identified who satisfied at least 1 of the 3 diagnosis codes. Among the 2,303 patients who did not meet diagnostic criteria for PTS, the majority (n = 1,784; 77.5%) were diagnosed with cervical spine–related pathology. Additional musculoskeletal conditions included rotator cuff involvement (n = 108; 4.69%) and non-specific shoulder pain (n = 155; 6.74%). Peripheral nerve–related conditions accounted for 90 cases (3.91%), followed by neuromuscular and movement disorders (n = 5; 0.22%) and bone disorders (n = 9; 0.39%). Eleven patients (0.48%) were categorized with other diagnoses, and 141 patients (6.13%) lacked sufficient diagnostic information. Following screening, there were 249 patients with confirmed PTS (Fig. 1).

### Patient demographics

**Figure 2:**
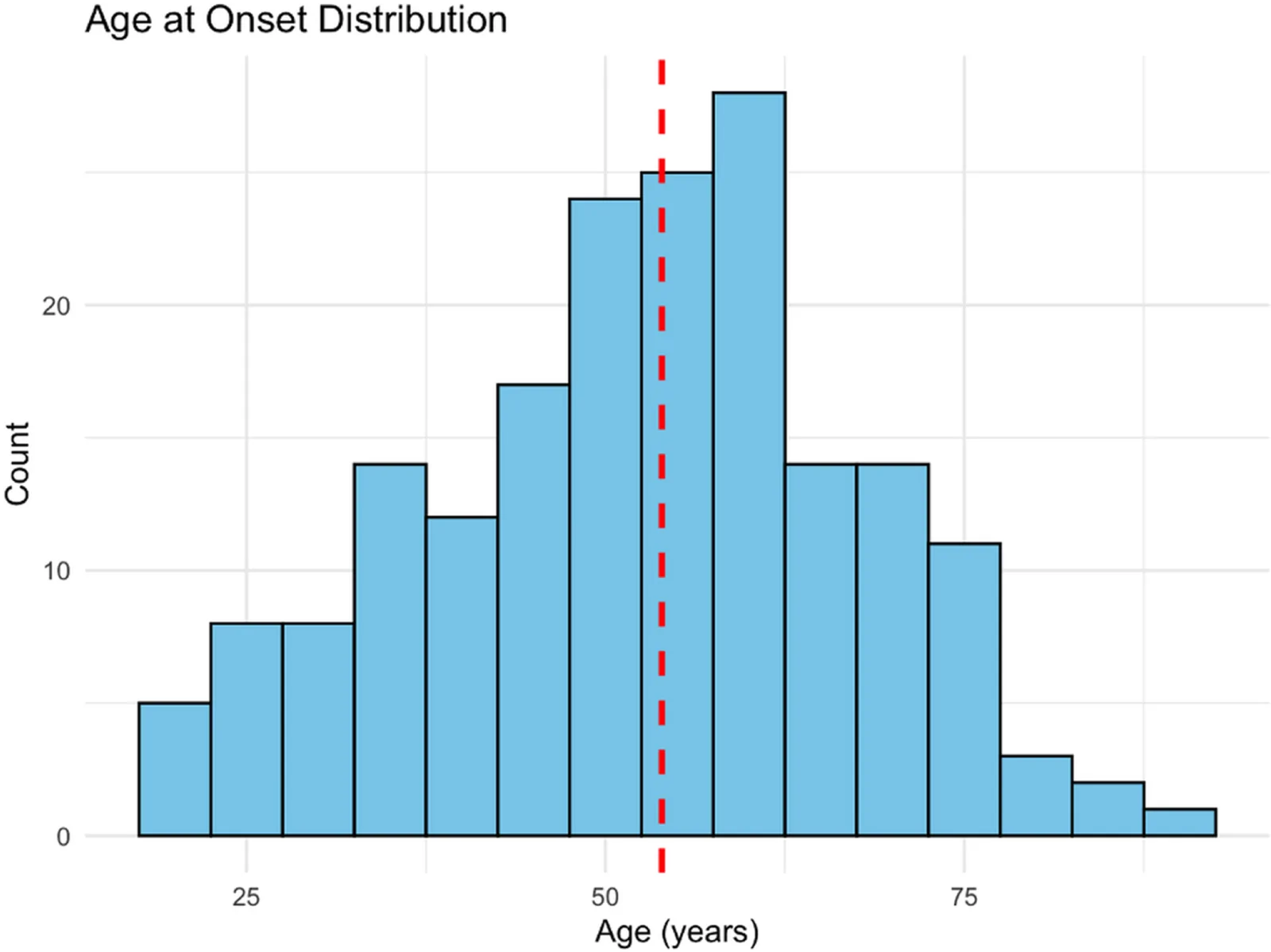
Age distribution of patients with Parsonage–Turner syndrome at symptom onset.

A total of 249 patients met inclusion criteria composed of 150 males (60.2%) and 99 females (39.8%). The median age at symptom onset (Fig. 2) was 54.2 (range: 19.1-88.6 years). Median age of onset was calculable for 186 patients with both a documented date of birth and a date of onset.

### Diagnostic classification

Diagnostic coding varied across patients. In the initial cohort of 2,552 patients, BN was the most frequently assigned diagnosis (n = 2,146), followed by neuralgic amyotrophy (NA) (n = 214) and PTS (n = 99). Seventy-seven patients carried dual diagnoses of PTS and NA, while 11 patients were coded as BN and NA and 3 were coded as BN and PTS. Two patients were assigned all 3 diagnostic codes.

In the confirmed PTS cohort, NA was the most frequently assigned diagnosis (n = 93), followed by PTS (n = 66) and BN (n = 13). PTS had the highest confirmation rate with 66.7% of original patients having confirmed PTS, followed by NA with 43.5%, and lastly by BN with 0.6%. Sixty-six patients carried dual diagnoses of PTS and NA, while 8 patients were coded as BN and NA, and 2 were coded as BN and PTS. One patient was assigned all 3 diagnostic codes concurrently.

### Hand dominance

Dominant hand status was available for 216/249 patients (86.7%). The majority were right-hand dominant (182/216, 84.3%), while 32 patients (14.8%) were left-hand dominant and 2 patients (0.9%) reported ambidexterity or mixed dominance. Dominant hand data were not documented for 33 patients (13.3%).

The side of clinical involvement was documented for 240/249 patients (96.4%). Among these, 121 patients (50.4%) presented with right-sided involvement, 89 patients (37.1%) with left-sided involvement, and 30 patients (12.5%) had bilateral disease. Among right-hand dominant patients (n = 182), 90 (49.5%) had ipsilateral right-sided involvement, 72 (39.6%) had contralateral left-sided involvement, and 20 (11%) had bilateral symptoms. Among left-hand dominant patients (n = 32), 11 (34.4%) had ipsilateral left-sided involvement, 17 (53.1%) had contralateral involvement, and 4 (12.5%) had bilateral symptoms (Table I). Counting bilateral disease as dominant arm involvement, dominant arm symptoms were present in 125 patients (125/214, 58.4%) out of 214 patients with dominant hand status availability (excluding 2 with ambidextrous dominance). When restricted to only unilateral cases (n = 190), dominant arm symptoms were present in 101 patients (53.2%).

**Table I:** Dominant hand and side affected.

Variable	N value (%)
Right dominant hand	182 (84.3)
Left dominant hand	32 (14.8)
Ambidextrous dominant hands	2 (0.9)
Right side affected	121 (50.4)
Left side affected	89 (37.1)
Both sides affected	30 (12.5)

### Potential triggers and etiologies

Potential triggers were recorded in (208/249) patients. All triggers and etiologies were sorted into one of the following categories: idiopathic, viral illness, viral vaccination, trauma, surgery, hereditary, and autoimmune. Fifty-seven point two percent (119/208) were classified as idiopathic. Among the identified triggers, vaccination was noted in 14.4% (30/208), viral illness in 8.2% (17/208), trauma in 12.5% (26/208), surgery in 4.3% (9/208), hereditary 1.9% (4/208), and autoimmune in 1.4% (3/208). Those marked as hereditary carried the SEPT9 mutation and none met a functional or 100% recovery more than once (Table II).

**Table II:** Triggers and etiology of Parsonage–Turner syndrome cases.

Demographics	Hazard ratio (HR)	95% CI LL	95% CI UL	*P* value
Male	0.99	0.57	1.72	.964
Age (ref: 18-30)				
31 to 45	1.11	0.38	3.23	.850
46 to 65	0.97	0.37	2.55	.958
>65	0.63	0.20	2.00	.432

*CI*, confidence interval; *LL*, lower limit; *UL*, upper limit.

### Diagnosis delay

Dates of both symptom onset and date of diagnosis were available for 162 patients. Due to the non-normal distribution of diagnosis delays, median was selected to best measure the central values. In this cohort, the median diagnosis delay was 89 days with a range of 3 to 982 days (2.69 years).

### Secondary testing

Diagnostic modality data were available for 184 of 249 patients (73.9%), after excluding 65 patients (26.1%) with incomplete or unspecified documentation. EMG was the most frequently used study and served as the sole diagnostic tool in 85 of 184 patients (46.2%). EMG performed in combination with MRI was recorded in 55 of 184 patients (29.9%). MRI alone was obtained in 20 of 184 patients (10.9%), and in 24 of 184 patients (13%), the diagnosis was made clinically without confirmatory advanced testing or imaging.

### Treatments

Physical therapy or occupational therapy (PT/OT) was the most common intervention, provided to 154 of 249 patients (61.8%). Of these 154 patients, 60 patients (39%) received PT/OT alone, while 94 patients (61.0%) underwent PT/OT in combination with other modalities such as medications or injections.

Pharmacologic therapy was documented in 95 of 249 patients (38.2%), most frequently consisting of oral corticosteroids (47 patients; 49.5%), gabapentin for neuropathic pain (39 patients; 41.1%), and non-steroidal anti-inflammatory drugs (NSAIDs) (29 patients; 30.5%). Less commonly used medications included muscle relaxants in 8 patients (8.4%).

Injection therapies were reported in 33 of 249 patients (13.3%). Surgical or procedural intervention was rare, occurring in 4 patients (1.6%).

### Specialist group differences

Treatments were stratified by the specialty overseeing the patient's care. All patients were categorized into groups based on the specialty of their treating physician. These groups were internal medicine, neurology, orthopedics, and physical medicine and rehabilitation. Non-surgical subspecialties including neurology, physical medicine and rehabilitation, and internal medicine were grouped together and compared to the surgical group made up of orthopedic physicians. Fourteen patients were referred to neurosurgery for diagnosis confirmation, but their care was under the treating neurologist and were thus grouped in the non-surgical group. All comparisons identifying specialty related differences are descriptive and highly susceptible to referral bias and are thus only presented as illustrative rather than a definite difference.

### Treatments by specialty

**Figure 3:**
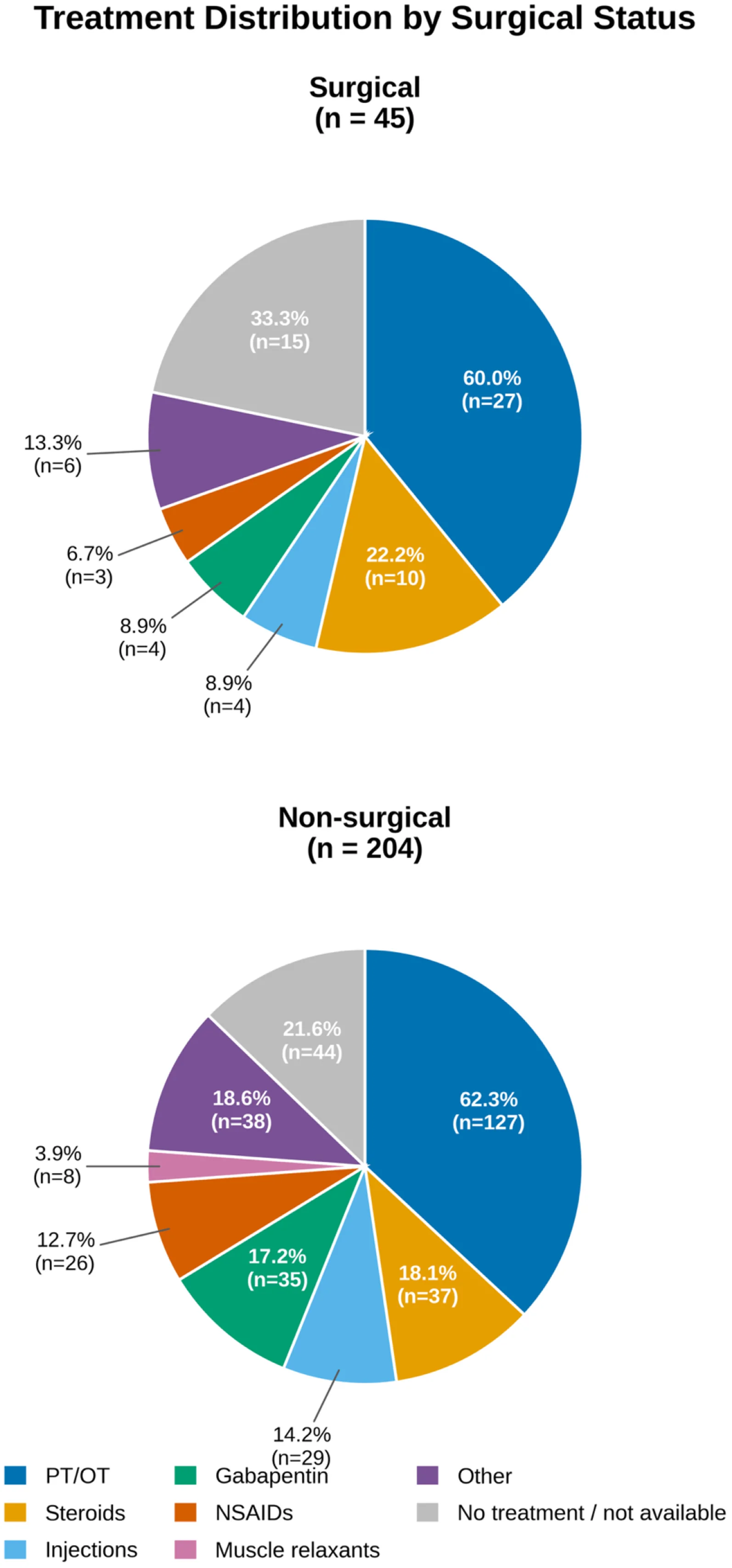
Specialty-specific distribution of treatment interventions among PTS patients. *PTS*, Parsonage–Turner syndrome.

Treatment interventions were categorized into 7 groups: PT/OT, gabapentinoids, steroids, muscle relaxants, injections, NSAIDs, and an “other” category encompassing miscellaneous or unclear interventions (Fig. 3).

### Surgical (n = 45)

PT/OT was prescribed in 27 patients (60.0%), followed by steroids in 10 (22.2%), gabapentin in 4 (8.9%), injections in 4 (8.9%), and NSAIDs in 3 (6.7%). Six patients (13.3%) received “other” interventions and 15 patients (33.3%) had no reported treatments.

### Non-surgical (n = 204)

PT/OT was prescribed in 127 patients (62.3%), followed by steroids in 37 (18.1%), gabapentin in 35 (17.2%), injections in 29 (14.2%), NSAIDs in 26 (12.7%), and muscle relaxants in 8 (3.9%) cases. Thirty-eight patients (18.6%) received “other” interventions and 44 (21.6%) had no reported treatments.

### Censored patients

**Figure 4:**
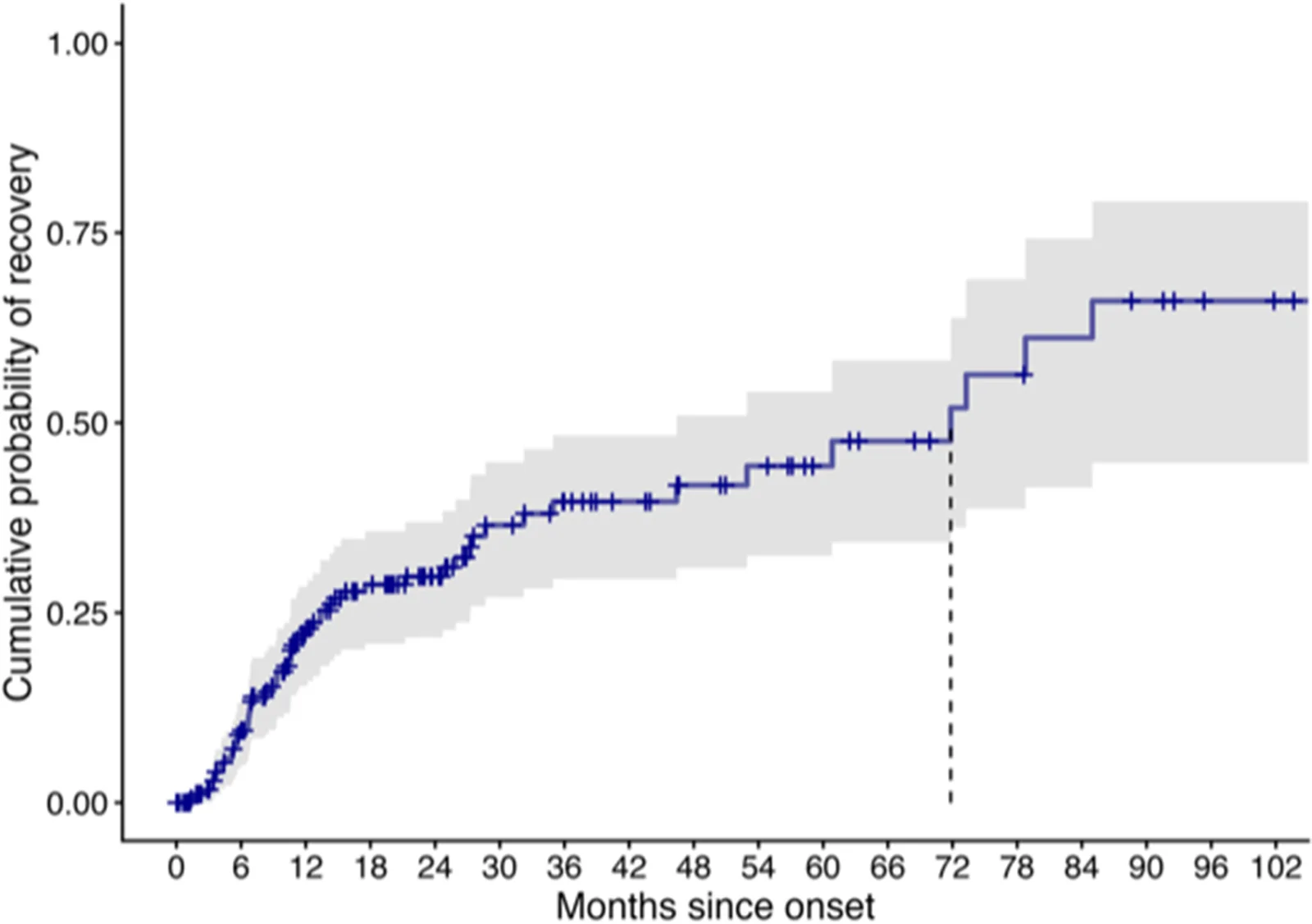
Cumulative censoring distribution.

Patients were followed from initial symptom onset to recovery or last contact. Those without an observed recovery were censored at last contact (eg, transfer of care or loss to follow-up). This approach allowed inclusions of patients who did not have a documented recovery in time-to-recovery analyses despite incomplete follow-up. The cumulative censoring patterns and the censoring patterns stratified by sex were right-skewed (Fig. 4, Supplementary Fig. 1, Supplementary Fig. 2). Most censoring occurred soon after symptom onset, reaching 50% at 11.7 months. This is followed by further censoring throughout the first 30 months following the onset of symptoms with a long, low-frequency tail extending beyond 90 months; 75% of censoring occurred by 25.6 months. Median follow-up was 11.2 months, and the reverse Kaplan–Meier estimate of median follow-up was 16.7 months. Males and females (Supplementary Fig. 2) mirror the initial pattern of censoring without evidence of a meaningful difference.

### Median survival

**Figure 5:**
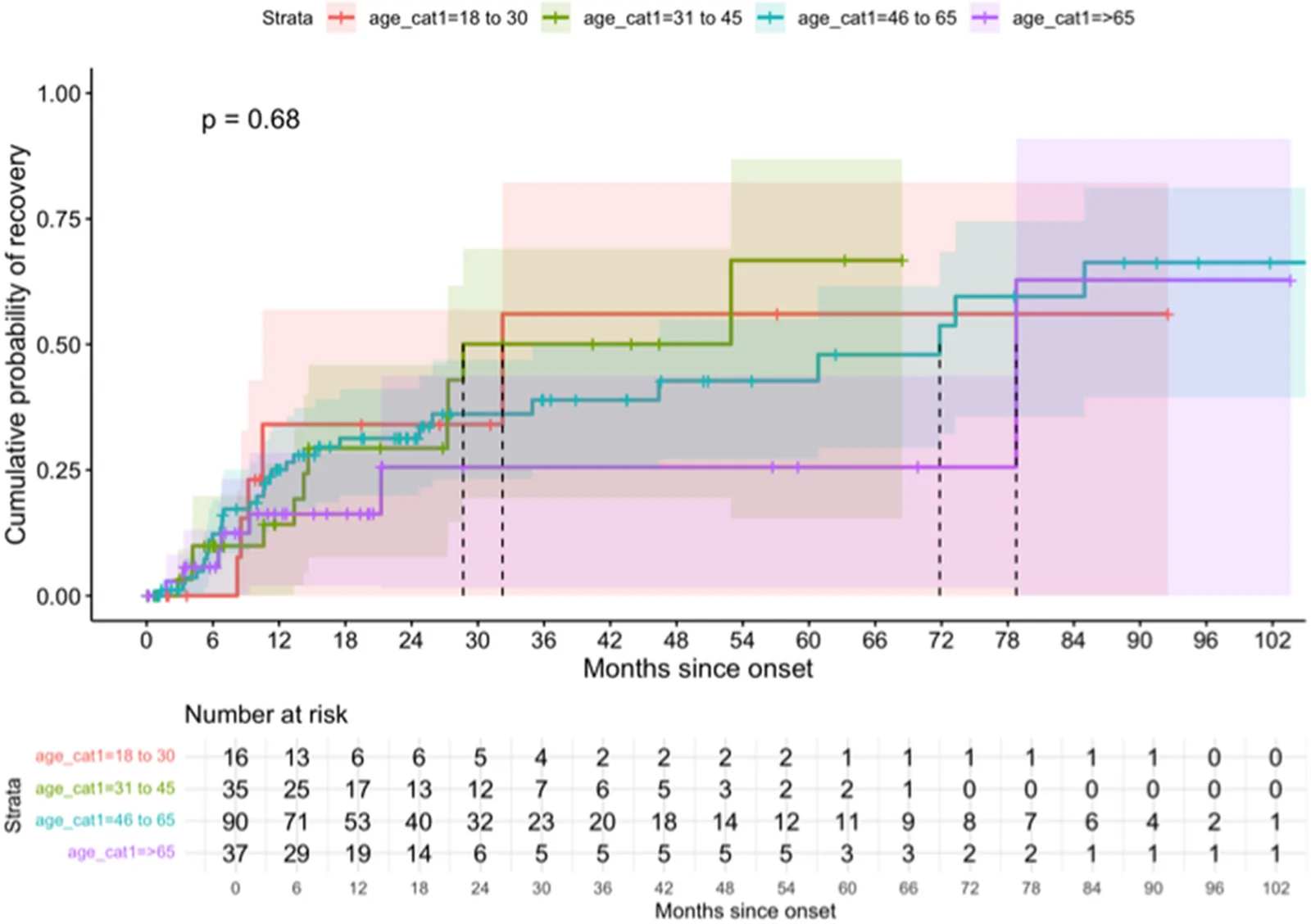
Probability of having achieved minimum functional recovery each month since symptom onset.

A Kaplan–Meier curve for the 216 PTS patients showed a rapid rise in recovery probability during the first twelve months, reaching approximately 22%, and then increased gradually until an estimated median time to recovery of 72 months (Fig. 5). Table III provides cumulative recovery probabilities from 12 to 60 months. The probability of recovery at 12 months was 22% (95% CI 15% to 28%), which increased to 44% (95% CI 32% to 53%) at 60 months.

**Table III:** Kaplan–Meier cumulative recovery probabilities.

Trigger/etiology	n	%
Idiopathic	119	57.2
Vaccination	30	14.4
Trauma	26	12.5
Viral illness	17	8.2
Surgery	9	4.3
Hereditary	4	1.9
Autoimmune	3	1.4
Total	208	100.0

### Median survival by age group

**Figure 6:**
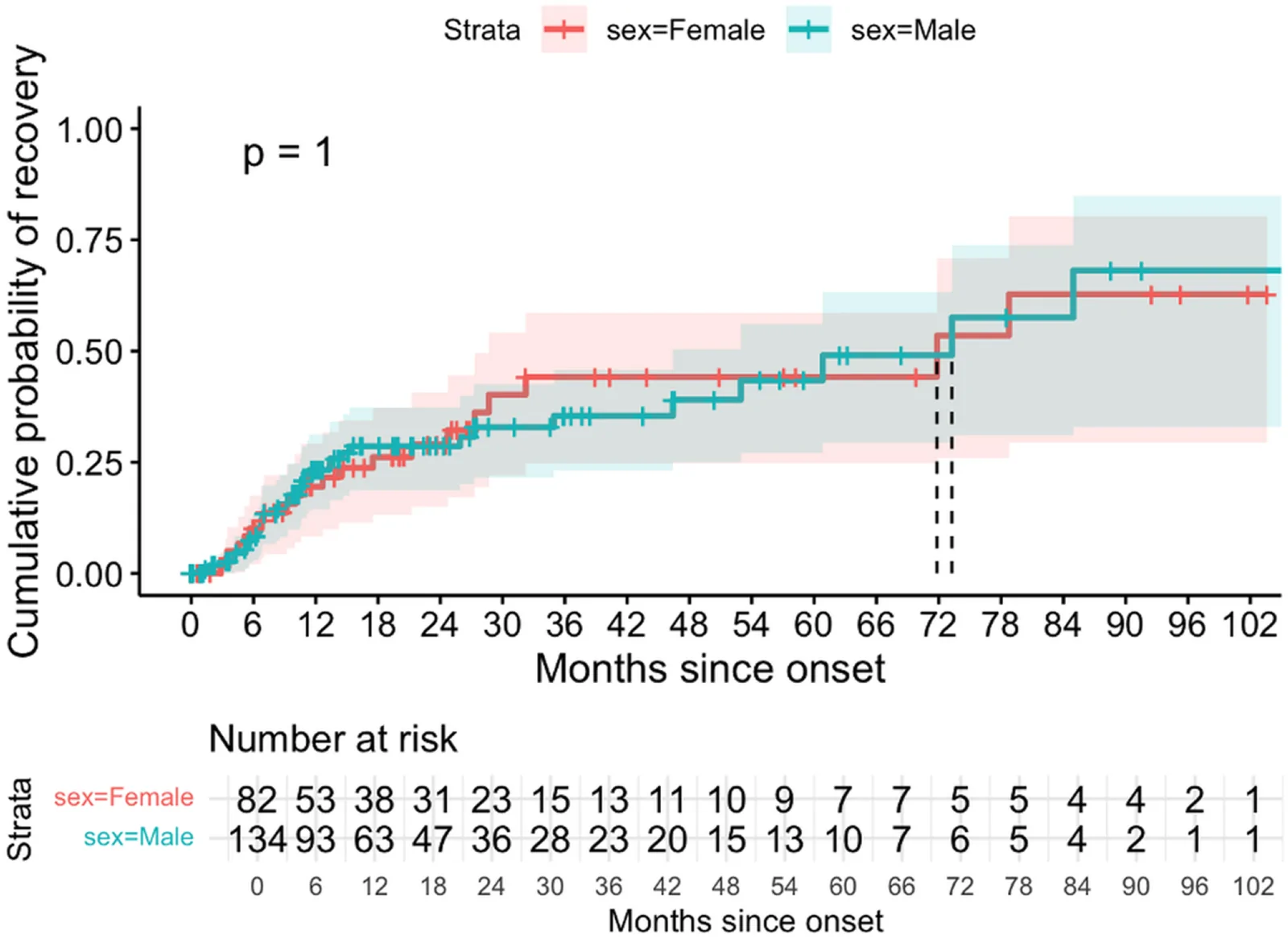
Cumulative Survival probability over time since symptom onset stratified by age group.

Patients were divided into 4 different age groups (18-30, 31-45, 46-65, >65 years). A Kaplan–Meier graph was created based on cumulative survival probability on the restricted cohort (n = 178), incorporating recovery and censoring data. Age groups did not affect recovery timeline (log-rank *P* = .68) (Fig. 6) (Table IV). Relative to the 18-30-year age group reference, adjusted hazard ratios (HRs) were 1.11 (95% CI 0.38-3.23) for the 31-45-year age group, 0.97 (95% CI 0.37-2.55) for the 46-65-year age group, and 0.63 (95% CI 0.20-2.00) for the >65-year age group, none reaching significance (Table IV).

**Table IV:** Cox proportional hazard model.

Kaplan–Meier cumulative recovery probabilities				
Months	Recovery probability	95% CI LL	95% CI UL	N at risk
12	22%	15%	28%	101
24	29%	21%	36%	59
36	39%	29%	48%	36
48	41%	30%	50%	25
60	44%	32%	53%	17

*CI*, confidence interval; *LL*, lower limit; *UL*, upper limit.

### Median survival by sex

**Figure 7:**
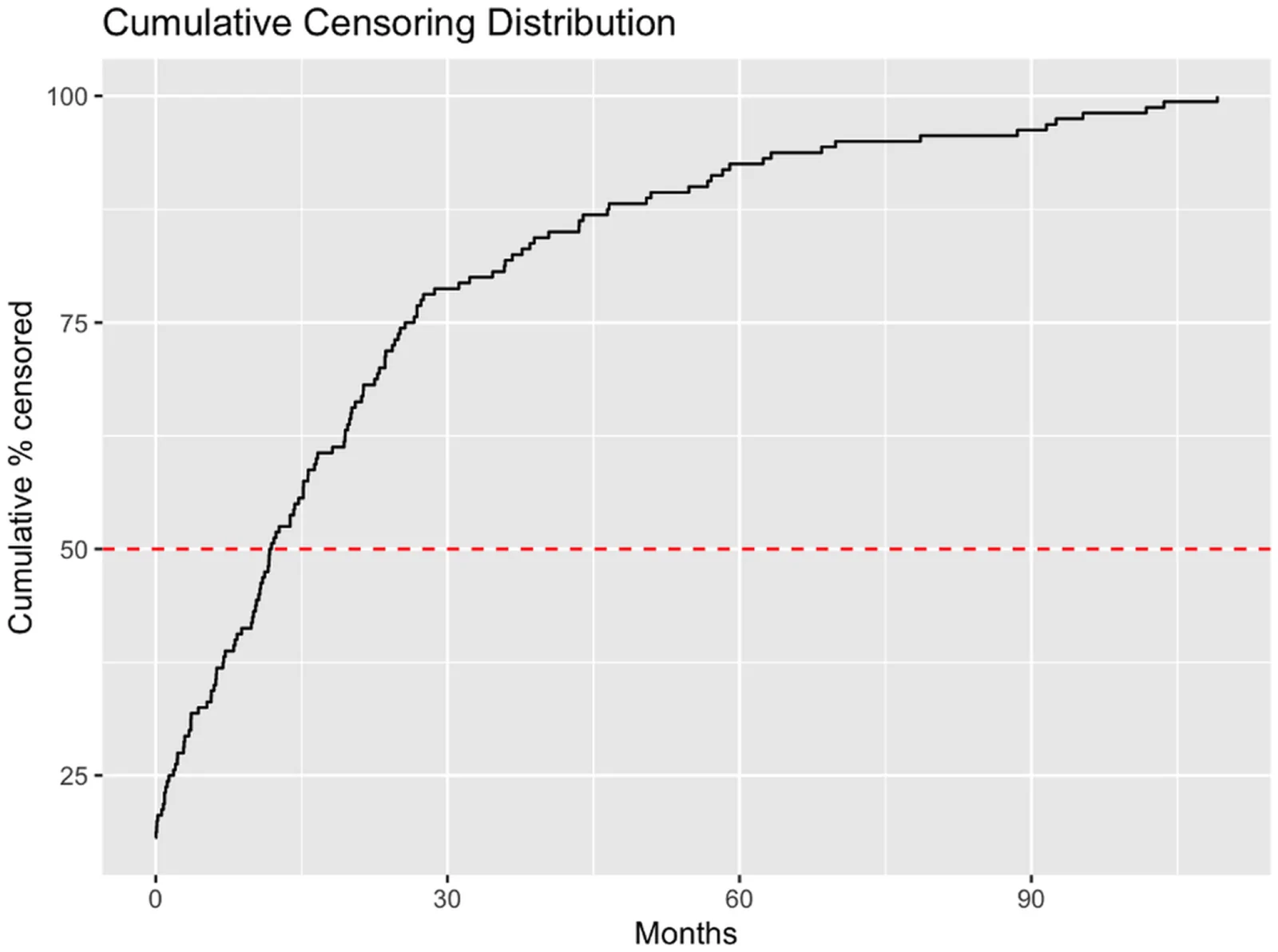
Probability of functional recovery by months since symptom onset, stratified by sex.

When stratified by sex (female n = 82, male n = 134), both curves tracked similarly through 60 months where most recovery and censoring occurred (Fig. 7). Sex did not affect recovery timeline (log-rank *P* = 1) (Table IV).

### Association of age and sex with recovery

Age and sex adjusted HRs for recovery are presented in Table IV. Sex was not associated with recovery in the Cox model (male, adjusted HR 0.99, 95% CI 0.57-1.72) (Table IV). When recovery was stratified by age in the restricted cohort (n = 178), age groups did not affect recovery timeline (log-rank *P* = .68) (Fig. 6) (Table IV).

### Results of patients with documented recovery

Data analysis of the recovered patient cohort (n = 56) was conducted to determine the median time to functional recovery, 100% recovery, and the time interval between both groups. Of the 56 patients with recovery data, 45 patients had functional recovery data, 19 patients had 100% recovery data, and 8 patients had both.

### Functional recovery

Among 45 patients with documented functional recovery milestones, the median time from symptom onset to functional recovery was 307.5 days (0.85 years) with an interquartile range (176.5 to 663). The range spanned from 39 days to 2,549 days.

### Hundred percent recovery

Complete recovery data were only available for 19 patients, likely reflecting the difficulty of capturing long-term outcomes from patients in a retrospectively designed study and these results should therefore be considered exploratory. The median time from onset to full recovery was 400 days (1.1 years), interquartile range 203 to 759, ranging from 90 to 2,555 days.

### Interval between functional recovery and 100% recovery

In the subset of 8 patients with both functional and 100% recovery dates, the median interval between these milestones was 170 days (0.47 years).

## Discussion

There is a lack of comprehensive literature investigating the outcomes of PTS patients. The complexity and rarity of the disease leave clinicians with little guidance in the identification and course of the disease, compounded by frequently misdiagnosed or undiagnosed cases. All data within this cohort were extracted from patient notes using Epic (Epic Systems Corporation). Although standardized guidelines were used to include patients in this dataset, variability among patient notes, imaging ordered and documented, and extractors exist. To combat this variability, every patient was assigned to 2 independent extractors, and when possible, a positive PTS case was confirmed using multiple clinical tools (patient history, physical exam, and imaging). Despite efforts to standardize patient selection, variability persists in this cohort.

This study found right-sided involvement (50.4%) more common than left-sided involvement (37.1%). Dominant arm symptoms were present in the majority of cases (53.2% of unilateral cases; 58.4% when bilateral involvement was included). This supports existing literature that generally shows the dominant side is preferentially affected,^25^^,^^32^ although largely in small sample sizes. The substantially larger cohort in this study further emphasizes this dominant arm predominance. Males (n = 150) were more commonly affected than females (n = 99) in a 3:2 ratio, echoing previous findings.^34^ Generally, PTS can either exist idiopathically or hereditarily.^44^ In the cohort studied in this paper, idiopathic (57.2%), vaccination (14.4%), trauma (12.5%), viral illness (8.2%), postsurgical (4.3%) were among the most common triggers. An electrodiagnostic lab has noted similar triggers with 54% of their cohort having an idiopathic trigger and 30% had a postsurgical trigger.^22^ Abdulsalam et al^1^ described a case of neuralgic amyotrophy developing immediately after needle EMG, underscoring the broad range of mechanical or inflammatory triggers capable of initiating an attack. Viral illnesses that preceded symptom onset included monkeypox virus,^36^ hepatitis E infection,^30^ cytomegalovirus disease,^28^ and COVID-19 virus, which others have also identified as an antecedent event.^6^^,^^29^^,^^33^^,^^37^ Among vaccinations that preceded symptom onset were the COVID-19 vaccination,^3^^,^^7^^,^^15^^,^^27^^,^^38^^,^^39^^,^^51^ the monkeypox vaccination,^36^ the typhoid vaccination,^24^ and postexposure prophylaxis for human immunodeficiency virus.^13^ These antecedent events were also identified by van Alfen and van Engelen,^50^ who found infection, exercise, and surgery as the 3 most common events in their cohort of 246 patients. Although surgical intervention was uncommon in our cohort (4%), emerging evidence suggests that select patients may benefit from early operative management. Chim described a series of COVID-associated PTS cases in which early surgical decompression of inflamed or compressed brachial plexus elements resulted in significantly improved pain and motor recovery compared with delayed intervention. While surgery is rarely indicated, certain post-viral or compressive variants may warrant earlier specialist evaluation.^8^

Due to numerous similarly presenting pathologies and the rarity of PTS itself, there was a delay between symptom onset and diagnosis (89 days).

This general delay in diagnosis can often be attributed to PTS being confused with rotator cuff disease and cervical disc disease.^44^ This is supported by this study, where, among the 2,303 patients (of the initial 2,552) that did not have PTS, the majority had cervical spine involvement (77.5%), followed by general shoulder pain (6.7%), and rotator cuff involvement (4.7%). The large number of cervical pathology cases included in the original 2,552 patient cohort is a direct consequence of the extraction codes. Two of the 3 ICD codes (M54.10 and M54.12) are radiculopathy codes, leaving only G54.5 specific to PTS. These codes were used to minimize missed cases since PTS is frequently miscoded as cervical radiculopathy yet led to the low yield that followed. Future database studies relying solely on ICD codes should anticipate this low yield and implement strict diagnostic criteria as seen in this study to ensure only confirmed PTS cases are included.

The most common treatments for those with PTS were PT and OT (61.8%) and pharmacologic treatments (38.2%). Among the pharmacologic treatments, oral corticosteroids (49.5%) were the most common followed by gabapentin (41.1%) and NSAIDs (30.5%). Injection therapies were used in 13.3% of patients and surgical intervention was rare (1.6%). Chu et al^9^ reported substantial functional improvement in a patient treated with a structured multimodal rehabilitation program incorporating scapular stabilization, neuromuscular reeducation, and progressive strengthening, illustrating how tailored therapy may accelerate recovery during the lengthy rehabilitation phase characteristic of PTS. Further reports have shown that intensive exercises can be performed after the onset of PTS without any damaging effects.^10^ These findings align with previous work underlining the importance of timely corticosteroid administration^20^ and PT to strengthen the affected muscles^42^^,^^50^ for PTS patients.

Recovery was presented in 2 distinct forms in this study. The first was the median recovery timeline for all patients with documented recovery (functional recovery, n = 45; 100% recovery, n = 19). The second was a Kaplan–Meier sub-analysis of a restricted cohort (n = 216) instead of the full 249 patient cohort, as 33 patients lacked date of onset of PTS diagnosis, which is indicative of diagnosis and treatment by an out of organization provider. The median recovery timelines describe how quickly recovery occurred among patients who both achieved recovery and returned to their physician to document the recovery and were thus conditional on documentation. By this measure, median time to functional recovery was 307.5 days and median time to 100% recovery was 400 days. The Kaplan–Meier median of 72 months instead considered all 216 patients in the restricted cohort, treating recoveries as events and incomplete follow-ups as censored and is heavily influenced by the censoring pattern in this cohort. Specifically, the median time to recovery is the estimated number of months by which 50% of patients would be expected to have recovered, accounting for censoring. Because some patients were censored soon after the onset and patients with improvements often discontinue follow-up, censoring is unlikely to be independent of recovery and likely overestimates the median. The outcomes of the Kaplan–Meier estimates should therefore be interpreted as a reflection of follow-up limitations rather than a measure of biological recovery time, and the medians of the documented recovery cohorts as a more direct, yet conditional, on recovery occurring, that is, the median time to recovery given that recovery occurred. The disparity between observed recovery time in recovered patients and Kaplan–Meier estimate reflects the influence of censoring and small number at risk at extended follow-up times. Because the reverse Kaplan–Meier follow-up was just 16.7 months, the 72-month estimate fell well beyond the timeframe, over which patients were actually observed and is thus an extrapolation into a range where increasingly few patients remained at risk. To aid in interpretation, cumulative recovery probabilities in 12 months intervals were provided, which indicate a large number of recoveries occurred in early follow-up with an estimated 44% probability at 60 months.

Two major patient demographics investigated in this cohort were age groups and sex. Literature is mixed regarding the role of sex in nerve injury outcomes, with some suggesting sex-related differences in neuropathic pain experience,^19^^,^^26^^,^^31^^,^^40^ while others find no significant effect on neurologic recovery^14^; age, however, is a well-established predictor of nerve damage recovery.^18^ The present study evaluated whether our cohort supported these differences in outcomes and further stratified outcomes by sex and age. Stratified by sex in the restricted cohort (n = 216), males and females demonstrated a similar median recovery time (∼72-73 months; log-rank *P* = 1.0) (Fig. 7) that aligned with the cumulative median time to recovery of 72 months (Fig. 5). Neither age nor sex demonstrated evidence of a meaningful impact on outcomes with nearly all HR centered at 1, with the exception of the >65-year age group when compared to the baseline 18-30-year age group (HR = 0.63). However, the range of the CI was large (95% CI 0.2-2.00), indicating substantial uncertainty in the estimate (Table IV).

These findings parallel recovery variability reported in the literature. In a 950-patient systematic review, Al Hinai et al found that more than half of conservatively managed patients demonstrated no meaningful improvement, and long-term follow-up at 5–25 months revealed persistent neuropathic pain in 60% and incomplete motor recovery in 70% of cases. Notably, while 87% of patients were initially treated non-operatively, a subset (15%) ultimately required surgical intervention, including neurolysis, decompression, or nerve transfer. The review emphasized significant delays in presentation (1–24 months) and persistent gaps in referral pathways, stating that heterogeneity in outcomes may not only be biological but also reflective of inconsistent recognition and management of PTS.^2^ Other cohorts share these heterogeneous outcomes with 1 hand surgery study finding just 44% of patients achieved complete recovery averaging 10 months,^32^ broader reviews showing 80% recovery within 6 months to 3 years of symptom onset,^41^ another reporting that 80-90% of patients will recover within 2-3 years,^48^ and a systematic review reporting 25% of their cohort unable to work at 3 years.^21^

While the study provides a comprehensive analysis of diagnostic characterization and outcome measures in patients with PTS, several limitations related to study design must be considered. The retrospective nature of the analysis relies on Epic electronic health record data, which makes it difficult to precisely determine the timing of symptom onset and recovery, subsequently complicating estimation of recovery time. Additionally, the absence of EMG data in a subset of patients is a further limitation. With lengthy recovery periods, typically spanning more than a year, long-term follow-up is required to accurately track patient outcomes. However, patient attrition and loss to follow-up limit analysis, hindering the ability to comprehensively capture longitudinal data. Additionally, complete recovery was rare in this cohort. Of the original 249 patients, only 45 patients had reached functional recovery, and only 19 reached full recovery; thus, recovery documentation was very rare. Future prospective cohort studies are needed to address these limitations by establishing uniform diagnostic criteria, systematic follow-up periods, and outcome measures to ensure similar diagnostic treatment for each patient. Formal inter-rater reliability was not quantified as independent extractor data were not kept after conflict resolution. Future studies should maintain individual reviewer determinations to be able to run these reliability tests. Additionally, incorporating patient-reported outcomes and evaluating treatment strategies would help clarify optimal clinical management practices for PTS.

## Conclusion

PTS is a rare disease with variable recovery timeline. Median documented recovery among observed recovery events occurred substantially earlier (functional recovery = 307.5 days; 100% recovery = 400 days), whereas Kaplan–Meier survival analysis suggested longer recovery trajectories (cumulative = 72 months) that may have been influenced by censoring patterns. Neither age nor sex was associated with recovery time. These findings can further help inform physicians and patients during the course of PTS pathology.

## Disclaimers:

Funding: No funding was disclosed by the authors.

Conflicts of interest: Bassem Elhassan is a consultant for Arthrex Inc. and DJ Orthopedics and has received royalties from DJ Orthopedics. Augustus D. Mazzocca is a consultant for Arthrex Inc., a consultant for Restor3d, and has stock market actions of Restor3d. Any additional authors, their immediate families, and any research foundations with which they are affiliated have not received any financial payments or other benefits from any commercial entity related to the subject of this article.
