# Supplementary material for: Outcomes and recovery timelines of 249 patients with Parsonage–Turner syndrome from a major tertiary care center
Source: JSES Int. 2026 Jul 22;10(6):101764. doi: 10.1016/j.jseint.2026.101764 (PMC13573188; doi:10.1016/j.jseint.2026.101764)
Supplement: Supplementary file 1 [file mmc1.docx]

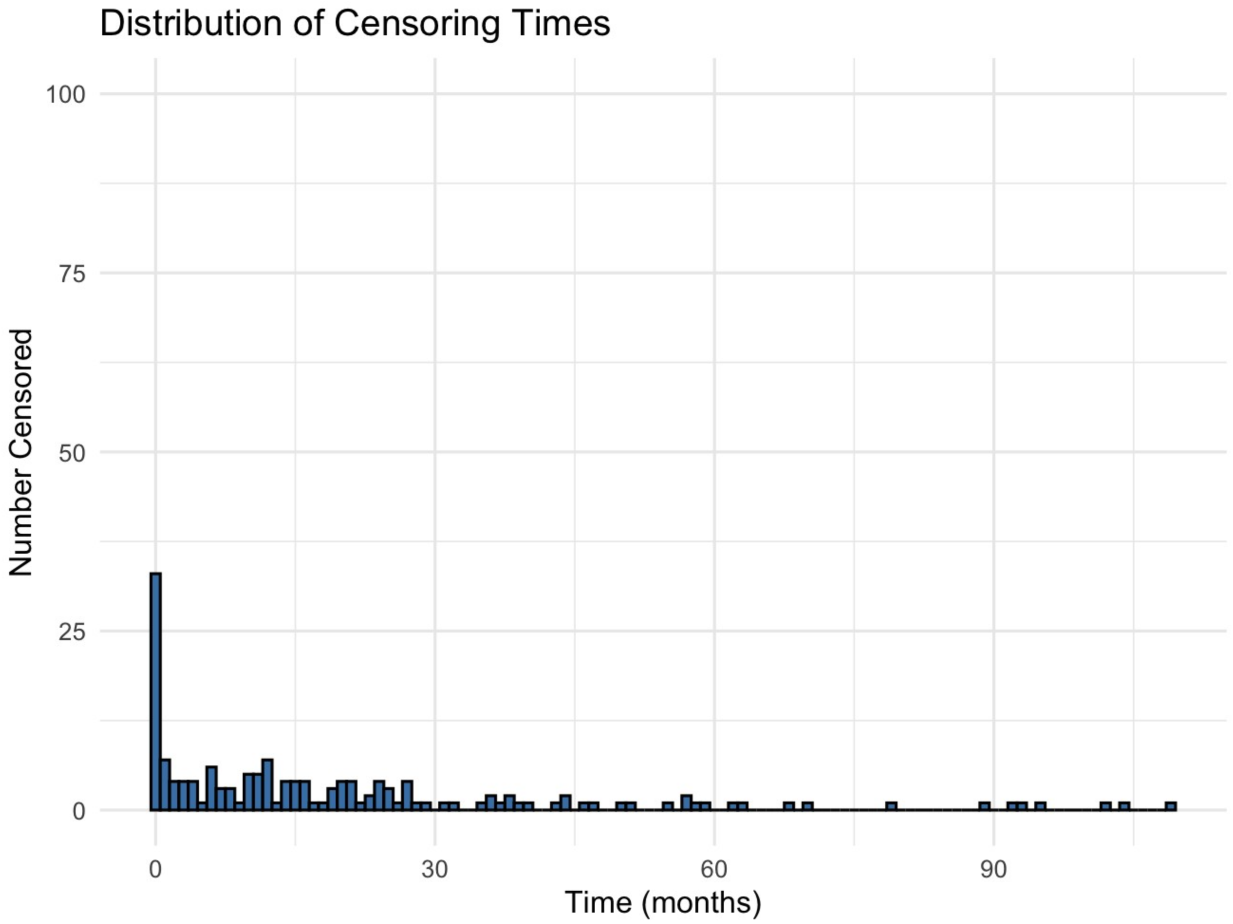

Distribution of Censoring Times
Number Censored
100
75
50
25
0
0
30
60
90
Time (months)
